# Naturopathic Care for Chronic Low Back Pain: A Randomized Trial

**DOI:** 10.1371/journal.pone.0000919

**Published:** 2007-09-19

**Authors:** Orest Szczurko, Kieran Cooley, Jason W. Busse, Dugald Seely, Bob Bernhardt, Gordon H. Guyatt, Qi Zhou, Edward J. Mills

**Affiliations:** 1 Division of Clinical Epidemiology, Canadian College of Naturopathic Medicine, Toronto, Ontario, Canada; 2 Department of Clinical Epidemiology and Biostatistics, McMaster University, Hamilton, Ontario, Canada; University of Ottawa, Canada

## Abstract

**Objective:**

Chronic low back pain represents a substantial cost to employers through benefits coverage and days missed due to incapacity. We sought to explore the effectiveness of Naturopathic care on chronic low back pain.

**Methods:**

This study was a randomized clinical trial. We randomized 75 postal employees with low back pain of longer than six weeks duration to receive Naturopathic care (n = 39) or standardized physiotherapy (n = 36) over a period of 12 weeks. The study was conducted in clinics on-site in postal outlets. Participants in the Naturopathic care group received dietary counseling, deep breathing relaxation techniques and acupuncture. The control intervention received education and instruction on physiotherapy exercises using an approved education booklet. We measured low back pain using the Oswestry disability questionnaire as the primary outcome measure, and quality of life using the SF-36 in addition to low back range of motion, weight loss, and Body Mass Index as secondary outcomes.

**Results:**

Sixty-nine participants (92%) completed eight weeks or greater of the trial. Participants in the Naturopathic care group reported significantly lower back pain (−6.89, 95% CI. −9.23 to −3.54, p = <0.0001) as measured by the Oswestry questionnaire. Quality of life was also significantly improved in the group receiving Naturopathic care in all domains except for vitality. Differences for the aggregate physical component of the SF-36 was 8.47 (95% CI, 5.05 to 11.87, p = <0.0001) and for the aggregate mental component was 7.0 (95% CI, 2.25 to 11.75, p = 0.0045). All secondary outcomes were also significantly improved in the group receiving Naturopathic care: spinal flexion (p<0.0001), weight-loss (p = 0.0052) and Body Mass Index (−0.52, 95% CI, −0.96 to −0.08, p = 0.01).

**Conclusions:**

Naturopathic care provided significantly greater improvement than physiotherapy advice for patients with chronic low back pain.

**Trial Registration:**

Controlled-Trials.com ISRCTN41920953

## Introduction

Non-specific chronic low back pain is a common cause of activity limitation in people younger than 45 years of age in developed nations and is one of the most common reasons for accessing physicians.[1] In addition to the burden of morbidity on individual patients, chronic low back pain is one of the most costly disorders for North American employers[2], [3].

In North America, non-specific low back pain is the most common reason for accessing complementary and alternative medicine (CAM) providers[4] with as many as 43% of all back-pain patients accessing CAM providers for care[5]. One complementary therapy provider group that deals with many back-pain patients as primary treatment providers are Naturopathic physicians[6]. To date however, no clinical trial has been conducted to evaluate the effectiveness of Naturopathic care for the treatment of low back pain. To inform this issue, we conducted a randomized controlled trial evaluating Naturopathic care versus a standardized physiotherapy education regimen for chronic low back pain.

## Methods

The protocol for this trial and supporting CONSORT checklist are available as supporting information; see Checklist S1 and Protocol S1.

### Study Design

This study was conducted from March to September 2005 at the Gateway Processing Plant of the Canada Post Corporation in Mississauga, Ontario- the largest processing plant in Canada. Canada Post employees who are members of the Canadian Union of Postal Workers (CUPW) were recruited through poster advertising at the plant and local depots. Interested employees received an information package which included a sample informed consent form, background information explaining the purpose of the study, a description of the intended naturopathic care, a question and answer sheet regarding study participation, and contact information for study enrolment. Workers were primarily from the Gateway plant, however a minority of the study population were from other Canada Post facilities. Two licensed Naturopathic physicians on site provided delivery of care (OS, KC). The institutional review board of the Canadian College of Naturopathic Medicine, in discussion with the Canada Post Corporation and CUPW, approved the study protocol.

All potential study participants were required to provide informed consent and to undergo a 1-hour assessment with a medical physician. Participants were evaluated for non-specific back pain through a thorough physical examination and completion of the Oswestry Low Back Pain Disability Questionnaire and the Roland and Morris Low Disability Questionnaire. Participants had to have had low back pain of non-specific cause for the preceding 6 weeks.

Participants were excluded if they could not comply with the study protocol, had mild or no pain at the time of assessment, a history of back surgery, sciatica, systemic or visceral causes of the pain, osteoporosis, a vertebral fracture or dislocation, severe neurological signs, spondylolisthesis, coagulation disorders, or a severe concurrent illness. Participants were also excluded if they were pregnant or were involved in claiming for compensation or litigation because of back injury. Use of pain medications was not a reason for exclusion.

This study was a randomized trial comparing Naturopathic care to standardized physiotherapy advice. The treatment interventions were planned for 12 weeks, with an option for control group participants to receive naturopathic care at the end of week 12 . The optional crossing-over period of treatment lasted 4 weeks. After participants were considered eligible and baseline information collected, they were randomized (1∶1) using double-observed coin-toss by OS and KC to either naturopathic care or an educational booklet. Although the investigators and data analysts were blinded to treatment allocation, it was not possible to mask the interventions from the patients or the clinicians delivering care.

### Treatment Groups

#### Naturopathic care

Participants receiving naturopathic care were seen twice per week to receive specific acupuncture treatment for low back pain, for a total of 24 treatments over a period of 12 weeks. Specific points needled were: GV 3,4, BL 23, 25, 40 bilaterally. Each needle was inserted 0.5 cun and needles were stimulated to achieve de qi (a dull sensation). Each needle was left in place for 20 minutes. The needles used were Seirin disposable needles number 5, 0.25×30 mm. Once needles were inserted the participants were instructed to perform diaphragmatic deep breathing exercises, and were counseled to consume a diet high in omega 3 fatty acids, magnesium and calcium. Participants were also encouraged to perform any kind of aerobic exercise, such as biking, walking, swimming, etc for 30 minutes 3 times per week.

#### Standardized Educational Booklet and Advice on Exercise and Relaxation Exercises

Participants randomized to the control group received an educational booklet, designed by the British Physiotherapy Association that has been previously validated to compare with active physiotherapy[7]. The booklet provided information on causes of back pain, prognosis, appropriate use of imaging studies and specialists, and exercises for promoting recovery and preventing recurrences. Participants receiving the information booklet were instructed to follow the general advice to remain active, as specified in the booklet. At each subsequent visit this group of participants received instruction on specific back stretching and strengthening exercises, and were educated about relaxation exercises.

### Outcomes

Our primary outcome was self-reported disability due to low back pain, as measured by the Oswestry Low Back Pain Disability Questionnaire, and Quality of Life, as assessed by the well-established Short Form 36. The Oswestry questionnaire characterizes the extent to which low back pain impacts on the participant's life, work, and daily function and is scored from 0 to 50. The higher the score, the more the low back pain affects his or her life: 0 to 10 (minimal disability), 11 to 20 (moderate disability): 21 to 30 (severe disability), 31 to 40 (crippled), and 41 to 50 (either bed-bound or exaggerating their symptoms. The SF-36 is a general quality of life indicator. The questionnaire aims to assess the degree to which specific quality of life measurements are affected by a course of treatment. It is not specific to low back pain, but rather measures the degree to which various aspects of the participants' life were affected by the treatment. Here a higher score indicates improvement in the particular quality of life category.

Secondary outcomes assessed included a self reported pain scale, the Roland Morris Disability questionnaire, forward lumbar flexion range of motion, weight, body mass index (BMI), use of non-steroidal anti-inflammatory Drugs (NSAIDS) and use paramedical interventions. All measures were assessed at baseline, week 4, 8 and 12. In order to assess the construct validity of the primary measurement tool for low back pain, we requested participants to complete the Roland Morris Disability questionnaire. Participants were asked about compliance, adverse events, and perceived benefit (Naturopathic care group only) at the same time periods. Compliance to the dietary recommendations was measured with the use of a diet diary, and through a checklist of questions about dietary intake at each visit.

Additionally, compliance with treatment was monitored on a semi-weekly basis using a percentage compliance scale, with <70% adherence considered non-compliant at each time point.

### Statistical analysis

All analyses were performed by a statistician (QZ) under blinded conditions using SAS/STAT (Version 8, Cary, NC). A sample of about 36 in each group was found to be adequate to detect a 10% change in the Oswestry Low Back Pain Disability Questionnaire assuming a between patient variability of 15% [8], [9], a two-sided significance level of 5%, and a power of 80%. A 5-point difference (10%) between groups has been established as the minimal clinically important difference.[9], [10]

Data were analyzed according to intention-to-treat. The means over the 12 week period were plotted for the outcomes of Oswestry, SF-36 physical component and SF-36 mental component separately. To assess the treatment effect for each group we calculated the mean change scores between groups at week 12 and the baseline. For any missing data at week 12, we carried forward the value at week 8. The statistical significance of the changes for each group was tested by the paired t-test and the exact 2-sided p-value is reported. To compare the change scores between groups, the two-sample t-test was performed.

The construct validity of the Oswestry questionnaires was evaluated in comparison to the Roland and Morris questionnaire. Our *a priori* assumption was good correlation between the 2 established questionnaires (>0.5). We used the Pearson Correlation Coefficient at baseline and at week 12 separately by active group and the control group.

## Results

### Recruitment and Follow-up of Patients

**Figure 1:**
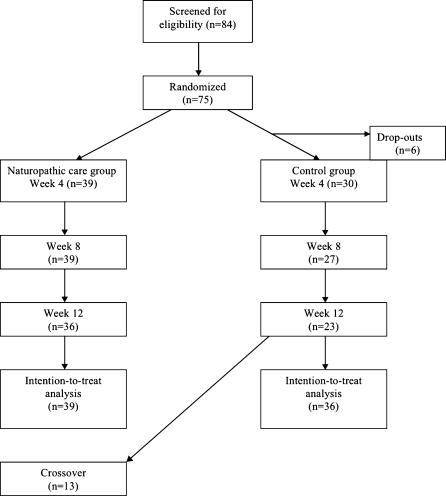
Flow diagram of participants through trial

We screened 84 participants eligible for the study. We excluded 9 participants for the following reasons: unable to commit to the study time commitments (n = 3); no lower back pain at time of assessment (n = 2); pregnant (n = 2); previous spinal surgery (n = 1); and, too late in accepting enrolment (n = 1). A total of 75 participants were enrolled into the study and randomized (39 to naturopathic care and 36 to the control intervention) and 63 completed the full study (Figure 1.). Of the 6 participants that dropped out, 5 were female, all were in the control group and all dropped out prior to assessing week 2 outcomes. The reasons cited for dropping out were: dissatisfaction with treatment (n = 3) and unable to commit to the time required (n = 3).

### Baseline characteristics

Table 1 displays the baseline characteristics of the groups. The majority of participants were internal workers with an appropriate make up of mixed ethnicities. The mean scores on the SF-36 general health perceptions subscale were below national norms[11]. All participants had had back pain for greater than six weeks that they reported as minimally disabling according to the scoring system of the Oswestry Low Back Pain Disability Questionnaire.

**Table 1:** Characteristics of participant groups.

	Naturopathic care	Control
Mean age (SD)	45.31 (7.46)	48.02 (8.27)
Percent women	56%	44%
Weight kgs (SD)	79.12 (14.39)	78.66 (18.13)
BMI	28.70 (4.87)	27.69 (3.68)
Number in shift	19 day	15 day
	8 letter carrier	4 letter carrier
	5 afternoon	7 afternoon
	5 night	9 night
	2 truck driver	1 truck driver
Percent day shift	48.7%	41.7%
White	22	17
Black	3	4
South Asian	11	12
East Asian	2	3
Aboriginal	1	0
Oswestry score	11.85 ( 8.18)	11.08 (7.83)
SF-36 Mental aggregate	−1.10 (0.86)	−1.19 (0.91)
SF-36 Physical aggregate	−0.27 (1.15)	−0.06 (1.18)
NSAID use, Median [range]	3 [0–49] (n = 21)	0[0–15] (n = 19)

### Study treatments

Data was available on 100% (39) of the naturopathic care group at week 8 and 75% (27) of the control group at week 8. Complete data on participants at week 12 was available on 92% and 63% respectfully.

Compliance was perfect for acupuncture and relaxation breathing techniques for participants randomized to naturopathic care. Compliance to dietary recommendations was excellent (87% of all visits, standard deviation [SD] 16%). Participants in the control group reported excellent compliance to the stretching and exercise interventions (81% of all visits, SD 28%). No important adverse effects were reported in either group.

### Outcomes

#### Primary Outcomes

**Figure 2:**
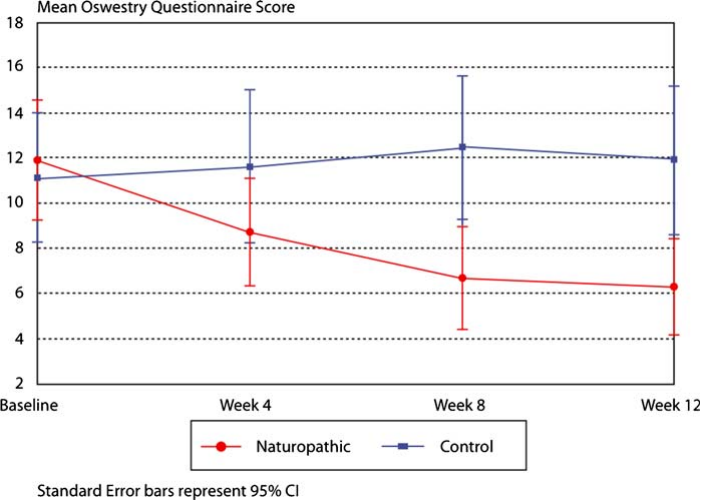
Oswestry questionnaire measuring disability over 12 weeks

**Figure 3:**
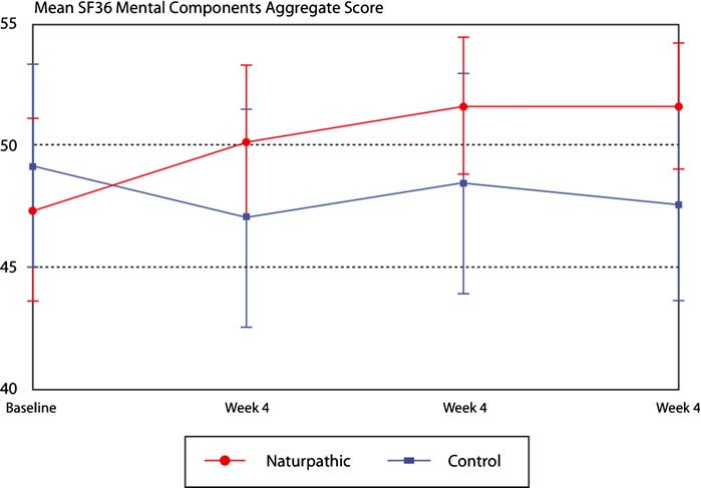
Mean SF-36 mental components aggregate over 12 weeks

**Figure 4:**
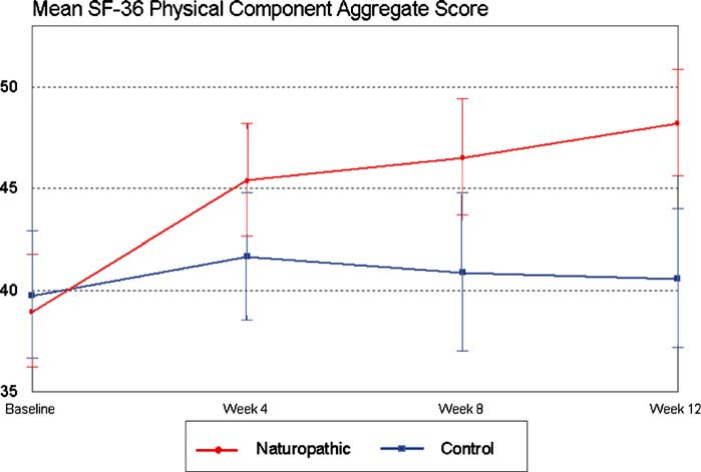
Mean SF-36 physical components over 12 weeks

The difference in mean change scores in the Oswestry Low Back Pain Disability Questionnaire from baseline to week 12 resulted in a significant reduction of disability in the naturopathic care group compared to the control group (median change = −5; p = <0.0001; Table 2). Figure 2 displays the mean scores for the Oswestry Low Back Pain Disability Questionnaire per group over the 12-week study period. SF-36 scores also displayed a significant difference between treatment groups for both the aggregate physical component (median change = 9.25; p = <0.0001) and the aggregate mental component (median change = 4.26; p = 0.0045) and for all domains except vitality (Table 3). Figures 3 and 4 display the mean SF-36 scores for the mental and physical aggregate components, respectively, over the 12-week study period.

**Table 2:** Comparisons for Oswestry, Roland and Morris, Pain Scale, Spinal Flexion, Weight and BMI

Outcomes	Group	Baseline Median (Q1–Q3)* (N)	Week 12** Median (Q1–Q3) (N)	Change at 12 wks from baseline Median (Q1–Q3) (N, P-value)	Difference of changes between groups Mean (95%CI) P-value
Oswestry^1^	Naturopathic	10 (5∼16) (39)	4 (1∼9) (39)	−5 (−7∼−2) (39, 0.0007)	<0.0001
	Control	9 (4∼16) (30)	12 (4∼16) (27)	0 (−2∼4) (27, 0.7126)	
Roland and Morris	Naturopathic	7 (3∼13) (39)	2 (0∼6) (39)	−4 (−7∼−2) (39, <0.0001)	<0.0001
	Control	5 (2∼7) (30)	8 (4∼10) (27)	2 (1∼−5) (27, 0.0613)	
Pain Scale (10-point)	Naturopathic	2 (1∼3) (39)	1 (0∼1.5) (39)	−1 (−1.5∼0) (39, <0.0001)	<0.0001
	Control	2 (1∼2) (30)	2 (1∼2) (27)	0 (0∼1) (27, 0.2585)	
Spinal Flexion (cm)	Naturopathic	30 (24∼34) (39)	34 (30.5∼38) (39)	4.5 (2.5∼7) (39, 0.0006)	<0.0001
	Control	31 (28∼34.5) (30)	30.5 (27.5∼33) (27)	−0.5 (−1.5∼0) (27, 0.3171)	
Weight (kg)	Naturopathic	79.12±14.39 (38)	77.61±14.02 (38)	−1.51 (−2.44, −0.58) (38, 0.0022)	−1.46 (−2.60, −0.32) 0.0052
	Control	78.66±18.13 (30)	78.83±18.21 (27)	−0.05 (−0.46, 0.36) (27, 0.8111)	
BMI	Naturopathic	28.70±4.87 (38)	28.12±4.47 (38)	−0.58 (−0.94, −0.22) (38, 0.0023)	−0.52 (−0.96, −0.08) 0.0106
	Control	27.69±3.68 (30)	27.74±3.68 (27)	−0.06 (−0.23, 0.12) (27, 0.5063)	

* The data is reported as Mean±Std if the sample is normally distributed. Q1 and Q3 are the 1^st^ quartile and the 3^rd ^quartile of the sample

** the week 8 value was used for week 12 If missing value was occurred at week 12.

1 Primary outcome measure

**Table 3:** Comparisons for SF-36 outcomes

Outcomes SF-36	Group	Baseline Mean±Std (N)	Week 12* Mean±Std (N)	Change at 12 wks from baseline Mean (95%CI) (N, P-value)	Difference of changes between groups Mean (95%CI) P-value
Aggregate physical component^1^	Naturopathic care	38.96±8.56 (39)	48.21±8.10 (39)	9.25 (6.81, 11.68) (39, <0.0001)	8.47 (5.05, 11.87) <0.0001
	Control	39.75±8.39 (30)	40.57±8.58 (27)	0.78 (−1.45, 3.02) (27, 0.4780)	
Aggregate mental component^1^	Naturopathic care	47.30±11.46 (39)	51.57±8.05 (39)	4.26 (0.88, 7.65) (39, 0.0149)	7.00 (2.25, 11.75) 0.0045
	Control	49.15±11.18 (30)	47.57±10.03 (27)	−2.74 (−5.86, 0.39) (27, 0.0835)	
Physical functioning	Naturopathic care	40.95±9.97 (39)	48.08±9.32 (39)	7.12 (4.69, 9.56) (39, <0.0001)	5.56 (1.93, 9.20) 0.0033
	Control	40.13±11.51 (30)	41.68±11.01 (27)	1.56 (−1.17, 4.29) (27, 0.2508)	
Role physical	Naturopathic care	40.97±10.88 (39)	49.63±9.25 (39)	8.67 (5.17, 12.16) (39, <0.0001)	11.48 (6.47, 16.49) <0.0001
	Control	42.57±9.94 (30)	40.98±9.66 (27)	−2.81 (−6.28, 0.66) (27, 1077)	
Bodily pain	Naturopathic care	36.80±7.71 (39)	47.92±7.88 (39)	11.12 (7.99, 14.25) (38, <0.0001)	10.83 (6.26, 15.40) <0.0001
	Control	38.76±7.51 (30)	39.21±6.48 (27)	0.29 (−3.00, 3.58) (27, 0.8578)	
General health	Naturopathic care	44.43±8.80 (39)	50.48±7.61 (39)	6.05 (3.31, 8.78) (38, <0.0001)	7.18 (3.58, 10.77) 0.0002
	Control	44.94±9.68 (30)	43.90±9.43 (27)	−1.13 (−3.02, 0.76) (27, 0.2310)	
Vitality	Naturopathic care	44.25±9.36 (39)	50.09±9.68 (39)	5.84 (2.58, 9.11) (38, 0.0009)	3.87 (−0.50, 8.25) 0.0814
	Control	46.16±9.92 (30)	48.74±8.87 (27)	1.97 (−0.53, 4.46) (27, 0.1177)	
Social functioning	Naturopathic care	41.75±11.36 (39)	50.70±9.04 (39)	8.95 (5.45, 12.45) (38, <0.0001)	10.57 (5.67, 15.47) <0.0001
	Control	46.12±9.97 (30)	44.93±11.12 (27)	−1.62 (−4.83, 1.60) (27, 0.3106)	
Role emotional	Naturopathic care	45.61±12.75 (39)	50.50±8.11 (39)	4.88 (0.70, 9.07) (38, 0.0234)	8.05 (2.08, 14.02) 0.0090
	Control	44.74±12.32 (30)	42.92±12.53 (27)	−3.17 (−7.26, 0.92) (27, 0.1236)	
Mental health	Naturopathic care	46.33±11.23 (39)	50.95±8.60 (39)	4.62 (1.84, 7.40) (38, 0.0018)	7.44 (3.58, 11.29) 0.0003
	Control	47.99±10.30 (30)	46.15±9.18 (27)	−2.82 (−5.27, −0.36 (27, 0.0260)	

Week 12*-the value at week 8 was used for week 12 If the missing value was occurred.

1 Primary outcome measure

#### Secondary Outcomes

Subjects receiving naturopathic care also demonstrated greater improvement in spinal flexion (mean change score = 5.49cm; 95% CI = 2.13 to 8.85), weight loss (mean change score = −1.46; 95% CI = −2.60 to −0.32), and BMI (mean change score = −0.52; 95% CI = −0.96 to −0.08) as compared to the control group (Table 2).

The use of NSAIDs for the treatment of pain was minor across groups. Participants in the Naturopathic care group reported a mean baseline use of NSAIDS as 7.23 pills per week (range 0–49) per week, while the baseline use of NSAIDs in the control group was 1.2 pills per week (range 0–15). NSAID use in the naturopathic group reduced to 0.5 after 12 weeks of treatment. The control group reported a mean change of NSAID use from 1.2 per week at baseline to 2.5 after 12 weeks of treatment.

### Optional Cross-over period

After completing the study, thirteen self-selected participants previously assigned to the control group crossed over to the naturopathic care intervention for a period of 4 weeks. After 4 weeks of naturopathic care, the mean Oswestry Low Back Pain Disability Questionnaire score of the crossover group was significantly reduced from week 12 (p = 0.0053) and was not different from the week 12 scores of the original naturopathic care group (p = 0.23). Aggregate mental and physical component scores of the SF-36 changed significantly within the crossover group after 4 weeks of care (p = 0.026 and p = 0.044) and were no longer different from the original naturopathic care group at week 12 (p = 0.53 and p = 0.24).

### Construct validity

Construct validity of the Oswestry questionnaire provided excellent correlation to the Roland and Morris questionnaire at baseline (0.72) and at week 12 in the Naturopathic care group (0.79) and control group (0.70).

## Discussion

For Canada Post employees with chronic non-specific low back pain, we found that naturopathic care was superior to a standardized educational booklet and advice on exercise and relaxation techniques in reducing reported disability, weight, and BMI, and in increasing the general quality of life and lumbar flexion of participants. To our knowledge, this is the first study to examine naturopathic care for chronic low back pain.

### Interpretation

Ours is not the first study to examine the role of acupuncture in treating low back pain[12]. It is however, the first study to combine acupuncture with relaxation techniques and dietary recommendations; a combination of treatment options reflecting naturopathic care. There are several strengths to consider when interpreting this trial. We used clearly defined entry requirements, randomized participants, and analyzed our data using intention-to-treat. As blinding of participants and providers was impossible due to the nature of the interventions, we blinded the analysts to group allocation. We contacted the participants who dropped out in order to determine their health status and reasons for dropping out. In addition, we gave the control group participants the option to cross-over at 12 weeks to receive naturopathic care. For the 13 participants (56%) who chose this option, their Oswestry Low Back Pain Disability Questionnaire scores and SF-36 scores improved significantly over a period of 4 weeks.

There are also several limitations to consider in this study. The naturopathic care group had a more active intervention than the control group; however, both groups were provided with advice on exercise and relaxation techniques and recent trials have found the educational booklet we used to be equivalent to routine active physiotherapy for the management of chronic low back pain [7], [13]. Despite the evidence supporting the effectiveness of this particular educational booklet, generally one could not assume the control intervention to be of equal benefit as that of an active intervention [14]. It may be, and is likely, that participants in the control group were aware that their intervention was a control intervention and so found their treatment less desirable. This likely explains why drop-outs were all from the control group. Our sample size was relatively small to detect small effects. However, we were appropriately powered to detect the large effects observed in the trial, as confidence intervals around the primary outcomes were precise. The lack of blinding of interventions is problematic, and indeed is a challenge in educational and physical manipulation trials. It is possible that certain sub-groups in the trial would benefit more or less from a particular treatment. We did not conduct sub-group analyses on our population as we believe our trial, and most trials, are underpowered to provide appropriate sub-group analyses [15], [16]. Further, whereas compliance to acupuncture and deep-breathing was directly observed by clinicians we relied on patient reported compliance to exercises and diet. It is possible that patient recall was insufficient. However, we did not expect nutrition to be the largest therapeutic effect and compliance to acupuncture and deep-breathing was directly observed by clinicians. Finally, in pragmatic randomized control trials such as this one, it is difficult to ascertain the non-specific or context effects of the treatments that were given.

### Generalizability

The generalizability of our study is limited as we enrolled participants from one large corporation and union. We believe however, that these conditions reflect a large number of corporate settings and clinicians and providers dealing with occupational health settings should find this study of interest. We additionally acknowledge that naturopathic care does vary according to practitioners and often includes different therapies than used in our study. Our naturopathic care intervention was designed in consensus with a team of academic naturopathic physicians to reflect current practices in Ontario.

### Overall Evidence

An important finding from this trial was the effectiveness of the Naturopathic care. We expected to see an effect on quality of life as the Naturopathic care group received treatment aimed at relaxation and improved nutrition. Quality of life appears to be linked to chronic back pain[1] and so our finding on quality of life improvement suggests that further research on the long-term effects of Naturopathic care would be of interest to patients. Further to this, we observed a significant decrease in the weight and BMI of participants in the Naturopathic care group compared to the control group, with a relative decrease of 1.46 Kgs (95% CI, −2.60, −0.32) between groups (P = 0.0052) and a mean decrease of 0.52 (−0.96, −0.08) BMI between groups (P for difference = 0.01).

Many patients access complementary therapy providers over physicians or more traditionally regulated professions due to the perception that CAM practitioners provide a more holistic treatment package, which examines physical complaints along with mental and emotional concerns, and indeed sometimes spiritual concerns[17]. Our study did not aim to determine participant contentment with Naturopathic care over other therapies, but we did receive systematic feedback in the form of optional comments on the forms that the participants in the Naturopathic care group increased their interest in seeking CAM care and developed an increased appreciation for Naturopathic care.

The results from this first randomized trial evaluating naturopathic care for chronic low back pain suggest that further research is warranted to determine the generalizability of this intervention, the specific contribution of individual treatment components, and the cost benefit associated with this therapy.

## Supporting Information

Protocol S1 Trial protocol
(0.05 MB DOC)

Checklist S1 CONSORT checklist
(0.06 MB DOC)
