# Supplementary material for: Naturopathic Care for Chronic Low Back Pain: A Randomized Trial
Source: PLoS One. 2007 Sep 19;2(9):e919. doi: 10.1371/journal.pone.0000919 (PMC1976391; doi:10.1371/journal.pone.0000919)
Supplement: Protocol S1 — Trial protocol (0.05 MB DOC) [file pone.0000919.s001.doc]

**Naturopathic Care for Low Back Pain: a Randomized Controlled Trial**

**Clinical Trial Protocol**

|  | **Name** | **Dept./Address** | **Phone No.** | **E-Mail** |
| --- | --- | --- | --- | --- |
| **Trial Design and Consulting** | Ed Mills  Dugald Seely | Research  Research | 416-498-1225 ext 324  416-498-1255 ext 387 | emills@ccnm.edu  dseely@ccnm.edu |
| **Research Co-ordinator** | Orest Szczurko | Research | 416-722-9136 | oszczurko@ccnm.edu |
| **Co-investigator** | Kieran Cooley | Research | 416-498-1255 ext 317 | kcooley@ccnm.edu |

Proposed Date of commencement: January 28, 2005

Location of Research: Gateway Canada Post Mail Processing Facility, 4567 Dixie Rd, Mississauga, ON

Rationale:

The Canadian Union of Postal Workers reports that diagnosed musculoskeletal complaints rose from 53% in 2001 to 59% in 2003. According to Canada Post Corporation's own research, they are experiencing escalating health care costs under their Extended Health Care Plan. Data from the research indicated that musculoskeletal injuries such as sprains, strains, slips, and falls were the most prevalent type of injury from 1994 to 2001 (Canada Post study). Physiotherapy, chiropractic, and massage services accounted for 83% of the total paramedical services used in 2003 (Canada Post study). The purpose of this study is to evaluate the efficacy of Naturopathic treatment on low back pain. This will also lay the groundwork for an assessment of the cost effectiveness of Naturopathic Medicine in the treatment of chronic low back pain in the work place.

Methodology:

Inclusion Criteria:

- mentally competent volunteers 18 to 65, male or female
- low back pain of more than 6 weeks duration
- normal on physical examination at the pre study physical

Exclusion Criteria:

- history of spinal operations
- current history of herniated disk
- current history of systemic rheumatological disease, ankylosing spondylitis, tumors, infection
- pregnancy

Setting and Participants: The setting for this study will be the Gateway Processing Plant of the Canada Post Corporation in Mississauga. The study will take place from January to June, and will involve Canada Post employees and members of the Canadian Union of Postal Workers (CUPW). Workers will primarily be from the Gateway plant, however a minority of the study population will be from other Canada Post facilities. There will be a mix of workers, from day to evening to night shift letter and package sorters, to outdoor letter carriers. The study population will be male and female, in the age groups from 18 to 65.

Sampling and Recruitment: Recruitment for the study will be facilitated by Canada Post Corporation and CUPW representatives. Posters and information sheets will be posted and distributed throughout eligible sites. Interested parties will be encouraged to contact the Naturopathic Doctors directly, either by phone or e-mail and provide their name, phone number, and mailing address. Interested participants will be mailed an Information Kit, which will include: a sample consent form, a Backgrounder explaining the purpose of the study, a description of Naturopathic Modalities, a question and answer sheet regarding the study, contact list and phone numbers of the Naturopathic Doctors, Canada Post, and CUPW representatives involved in the study, the Naturopathic Doctors business cards. Interested participants will be encouraged to ask questions to their Canada Post or CUPW representatives, and Naturopathic Doctors. Following the mailing of the Information Kit, interested participants will be contacted by telephone, and scheduled for a 1 hour initial consultation in a designated room at the Canada Post Gateway Processing Plant. At this consultation, any outstanding questions will be answered by the Naturopathic Doctors, a consent form will be signed and witnessed, and the intake interview and physical exam to evaluate the eligibility for the study, will take place. At the end of the initial consultation, participants will receive the Pain Medication and Pain Therapies Diary chart to complete at home over the next week. Eligible participants will be randomly assigned to either the control or treatment groups, and will be contacted by telephone regarding further appointment times.

At the first visit, prior to any treatments, the Oswestry, Roland and Morris, and SF-36 questionnaires will be completed by the patient to assess baseline. The Pain Medication and Pain Therapies diary will be collected. Both treatment and control groups will receive The Back Booklet, and patients will be instructed to follow the treatments as instructed in the booklet. On subsequent visits, the control group patients will be questioned on the performance of the Book Exercises, and will be encouraged to continue performing them.

The treatment group patients will be positioned prone, and specific acupuncture treatment for low back pain will be applied. During the acupuncture treatment, data on patients’ lifestyle and diet will be obtained, and diaphragmatic deep breathing exercises will be performed. On subsequent visits, the treatment group will continuously receive acupuncture treatment, receive lifestyle and diet counseling, continue to perform deep breathing relaxation techniques, and be encouraged to continue with the exercises as instructed in the exercise booklet.

Intervention arm patients will attend two treatments per week, for a total of 24 treatments over the period of 12 weeks. Control arm patients will receive one initial session with a Naturopathic Doctor, who will give them general advice and exercises as specified in the booklet, and will have bi-weekly visits with the Naturopathic Doctor who will question them on the performance of the Book Exercises, and will encourage the patient to continue performing them.

Both the Intervention and Control arm will complete the Oswestry, Roland and Morris, SF-36, and Perceived Benefit of Treatment questionnaires at 4, 8, and 12 weeks during the study. Both groups will also hand in the Pain Medication and Treatment diary chart, and exercise log at every visit.

Study period is 12 weeks. Clinical evaluations will be performed at:

Baseline (Visit 1 - Day 1)

Interim for the treatment groups: (Visit 2 - Day 7, Visit 3- Day 10, Visit 4 – Day 14, Visit 4 – Day 17, Visit 5 – Day 21, Visit 6 – Day 24, Visit 7 – Day 28, Visit 8 – Day 31, Visit 9 – Day 35, Visit 10 – Day 38, Visit 11 – Day 42, Visit 12 – Day 45, Visit 13 – Day 49, Visit 14 – Day 52, Visit 15 – Day 56, Visit 16 – Day 59, Visit 17 – Day 63, Visit 18 – Day 66, Visit 19 – Day 70, Visit 20 – Day 74, Visit 21 – Day 77, Visit 22 – Day 81, Visit 23 – Day 84, Visit 24 – Day 88, Visit 25 - 91) ie. Monday and Thursday, or Tuesday and Friday.

Interim for the control groups (Visit 2 – Day 7, Visit 3 – Day 21, Visit 4 – Day 35, Visit 5 – Day49, Visit 6 – Day 63, Visit 7 – Day 77, Visit 8 – Day 91)

End of Treatment (Visit 25 - Day 91), Questionnaires and Medication diaries filled in for the last time

Follow-up data will be collected at 6 months post treatment via a mailed questionnaire package containing the Oswestry, Roland and Morris, and SF-36 questionnaires, Perceived Benefit of Treatment scales, as well as the pain killer and adjunctive back pain treatment chart. Non-responders will receive reminders by post. If this is unsuccessful, the patients will be telephoned.

# Outcomes

Our primary outcome is self-reported low back pain measured by the Oswestry Low Back Pain Disability Questionnaire, and Quality of Life (SF 36). The Oswestry questionnaire characterizes the extent to which low back pain impacts on the participant’s life, work, and daily function and is scored from 0 to 50, or 0% to 100%. The higher the score, the more the low back pain affects life: 0% (no disability) to 100% (totally disabled or bed ridden). The SF-36 questionnaire aims to assess the degree to which specific quality of life measurements are affected by a course of treatment. The higher score indicates improvement in the particular quality of life category.

Secondary outcomes assessed will included self reported pain scale, the Roland Morris Disability questionnaire, forward lumbar flexion range of motion, weight, body mass index (BMI), use of non-steroidal anti-inflammatory Drugs (NSAIDS) and use paramedical interventions. All measures will be assessed at baseline, weeks 4, 8 and 12. We will assess the construct validity of the Oswestry with the the Roland Morris Disability questionnaire and assume good correlation (>0.5). We will ask for self-reported adherence to the regimen, adverse events, and perceived benefit (Naturopathic care group only) at the same time periods.

Adherence with treatment will be monitored on a semi-weekly basis using a percentage compliance scale, with <70% adherence considered non-compliant at each time point. This is a strategy we previously assessed in the HIV/AIDS field.

# Statistical analysis

Randomization will occur using blocked randomization (1:1) and will be conducted using double-observed coin-toss. In the event that the coin should fall, the face up side will be considered the allocation. Heads active, tails control. Participants will not be allocated to any group prior to sequence generation. All analyses will be performed by Qi Zhou PhD, a statistician, under blinded conditions. The sample size estimates indicate that a sample of about 36 in each group will be adequate to detect a 10% change in the primary outcome of self-reported pain assuming an intrapatient variability of 15%, a two-sided significance level of 5%, and a power of 80%.

All data will be analyzed according to intention to treat. We will calculate the mean change scores between groups at the specific weeks and the primary endpoint of change from base line to week 12. For any missing data at week 12, we will use LOCF at week 8. Paired t-tests will assess within group changes and two-sample t-tests will compare between groups. All p-values will be exact.

Participants

According to the sample size calculations, 75 participants, randomly allocated to treatment and control group, will take part in this study. Participants will be Canada Post employees, and CUPW union members. Most will be from Mississaugas' Gateway Processing Centre, with a minority coming from Canada Post centers from the surrounding area. Their job function will be varied, ranging from sorting workers to letter carriers. The age range will be workers from 18 to 65 years old.

Recruitment:

Recruitment for the study will be facilitated by Canada Post Corporation and CUPW representatives. Posters and information sheets will be posted and distributed throughout eligible sites. Interested parties will be encouraged to contact the clinicians directly, either by phone or e-mail and provide their name, phone number, and mailing address. Interested participants will be mailed a Participants Information Kit which will include: a sample consent form, a Backgrounder explaining the purpose of the pilot, a description of Naturopathic Modalities, a question and answer sheet regarding the study, contact list and phone numbers of Naturopathic Doctors, Canada Post, and CUPW representatives, Naturopathic Doctors business cards. Interested participants will be encouraged to ask questions to their Canada Post, CUPW, and Naturopathic Doctors. Interested participants will be contacted by phone, and scheduled for a 1 hour initial consultation in a designated room at the Canada Post Gateway Processing Plant. At this consultation, any outstanding questions will be answered by the Naturopathic Doctors, a consent form will be signed and witnessed, and the intake interview and physical exam will take place. Eligible participants will be randomly assigned to either the control or treatment groups, and will be contacted by telephone regarding further appointment times.

Choosing to withdraw will terminate any further study treatment. Withdrawal will not carry any negative consequences towards the participant from the Naturopathic Clinicians, CUPW or Canada Post Corporation. Participants in the control group that choose to begin treatment will be offered to begin treatment at the conclusion of the trial and we will follow them for an additional 4 weeks.

Risks:

Acupuncture carries with it a minimal risk of infection or organ perforation. All attempts will be made by the Naturopathic Doctors to ensure proper sterile and clean field acupuncture techniques, as specified in the BDDT acupuncture guidelines. Acupuncture points near organs which may be perforated will not be needled. Proper exercise procedures will be initially explained and demonstrated to the patient, but there remains a chance that improper exercise use may harm the patient.

Proper sterile acupuncture technique, as specified in the BDDT acupuncture guidelines, will be used. During the initial visit, potential subjects will be screened for risk factors. Patients will be provided with Naturopathic Doctors contact information, and will be encouraged to contact them with any questions, concerns, or emergencies. To minimize the risk of fainting associated with acupuncture, patients will be treated in a prone position.

Possible Benefits:

Participants may benefit by gaining permanent or temporary relief from their chronic back pain. They will gain an understanding of the process of research, by being active participants in it. The information provided by this study will allow for a better understainding of the role of Naturopathic Medicine in the workplace, and may lay the foundation for an in-depth evaluation of the cost / benefit analysis of Naturopathic Medicine in the work place.

The Consent Process:

Orest Szczurko or Kieran Cooley will telephone each person who volunteers to participate in the study to introduce him/herself, explain the purpose and scope of the study, and assure each individual of anonymity, both during the study and in published reports. The potential participants will be assured that they can stop participating in the study at any time without penalty. At the conclusion of the telephone call, arrangements will be made to mail the Participants Information Kit to interested parties. The kit will contain a sample consent form, Background information on the project, a description of Naturopathic Modalities, Question and Answer sheet, Contact list and phone numbers, and Naturopathic Doctors business cards. When provided with the study packet, they will be asked to read the material, and then direct any questions to either their CUPW or Canada Post representative, or Naturopathic Doctor. Interested parties will be asked to come for a 1 hour asessment visit at the Gateway site, which will first answer any further questions, and then sign the consent form if they still wish to participate in the study.

Confidentiality:

All patients will be assigned a unique number during the intial visit, all analysis will be performed using the patients assigned number. The records and questionnaires will be kept in a locked filing cabinet and shared only with study personnel. The records and questionnaires will be kept at CCNM for 25 years unless a patient requests otherwise.
